# A Rare Case of Salmonella Lung Abscess in an Immunocompromised Patient

**DOI:** 10.7759/cureus.111546

**Published:** 2026-06-26

**Authors:** Hanien Samara, Carl Cosue, Travis Martinez, Ankur Bhagat, Mufadda Hasan

**Affiliations:** 1 Internal Medicine/Pulmonary Critical Care, Western University of Health Sciences, Pomona, USA; 2 Internal Medicine, Arrowhead Regional Medical Center, Colton, USA; 3 Pulmonary and Critical Care Medicine, Arrowhead Regional Medical Center, Colton, USA

**Keywords:** case report, immunocompromised, lung abscess, pulmonology, salmonella

## Abstract

*Salmonella enteritidis* (*S. enteritidis*) is a Gram-negative, acid-labile bacterium responsible for major foodborne outbreaks in both developing and developed countries. While Salmonella species are often linked with gastrointestinal illness, pulmonary involvement is rare. We report a case of a Salmonella lung abscess occurring in the setting of Salmonella septicemia, in an immunocompromised patient with perinuclear anti-neutrophil cytoplasmic antibody (p-ANCA)-associated microscopic polyangiitis (MPA) and clear cell renal cell carcinoma (ccRCC). Initially admitted to Arrowhead Regional Medical Center (ARMC), Colton, for gastroenteritis secondary to Salmonella and *E.Coli* with subsequent bacteremia, our patient was readmitted for persistent weakness and diarrhea status post antibiotic treatment. He was found to have a 3.9 cm cavitary lesion within the left upper lobe on CT imaging, with subsequent biopsy wound culture confirming Salmonella lung abscess. Hospital admission was complicated by left popliteal vein deep vein thrombosis and native left hip septic arthritis with avascular necrosis. The patient was discharged following left hip washout and an eight-week course of ceftriaxone. Through this case, we hope to raise awareness of Salmonella-induced pulmonary disease within immunocompromised patients, as early recognition remains crucial for prompt resolution and prevention of severe complications.

## Introduction

*Salmonella enteritidis* is a Gram-negative, acid-labile bacterium responsible for major foodborne outbreaks in both developing and developed countries. Traditionally, Salmonella infections are associated with significant morbidity and mortality worldwide. It is estimated that approximately 93.8 million cases of gastroenteritis caused by Salmonella species occur globally each year, resulting in approximately 155,000 deaths [1,2]. Significant increases in disease morbidity/mortality are linked with disseminated Salmonella infection, with an estimated fatality of 20% (regardless of hospitalization) for non-typhoidal Salmonella bacteremia [3]. Risk factors for disseminated Salmonellosis include extremes of age, alteration of the endogenous intestinal bowel flora, diabetes mellitus, malignancy, rheumatological disorders, reticuloendothelial blockade (such as malaria, sickle-cell disease, or bartonellosis), HIV infection, and therapeutic immunosuppression [4]. 

Pulmonary manifestations of *S. enteritidis* infection are rare, with only a few cases describing patients with nonspecific respiratory symptoms (cough and dyspnea) in parallel with or immediately following a diarrheal illness. Definitive diagnosis of pulmonary S. enteritidis typically requires confirmation by blood or sputum cultures or by biopsy. Given the rarity of this condition, there are no standardized diagnostic or treatment guidelines, and management is often guided by case reports and expert opinion. 

Here, we present a rare case of an *S. enteritidis* lung abscess in an immunocompromised patient with perinuclear anti-neutrophil cytoplasmic antibodies (p-ANCAs) associated with microscopic polyangiitis and clear cell renal cell carcinoma. This case is noteworthy due to the presence of multiple risk factors for dissemination (including rituximab therapy, chronic corticosteroid use, and malignancy), as well as the presence of concurrent extraintestinal complications during admission (septic arthritis). This case highlights the diagnostic complexity of cavitary lung lesions in immunocompromised patients, where infectious and inflammatory etiologies may overlap. Identification of S. enteritidis as a possible cause of pulmonary cavitary disease is key to ensuring timely diagnosis and appropriate management.

## Case presentation

Initial presentation

Our patient is a 56-year-old man with a past medical history including diabetes mellitus type 2, p-ANCA associated microscopic polyangiitis (MPA) with interstitial lung disease (ILD)/pulmonary hypertension, severe obstructive sleep apnea (noncompliant with continuous positive airway pressure (CPAP)), left popliteal vein deep vein thrombosis (on eliquis), anemia of chronic disease, chronic obstructive pulmonary disease (COPD), chronic kidney disease (CKD) stage 4 with paci immune crescent glomerulonephritis, clear cell renal cell carcinoma of the right kidney, heart failure with preserved ejection fracture and 10 pack/year smoking history (quitting five years prior to admission). He was admitted to our hospital following a mechanical fall secondary to weakness, in the setting of Salmonella gastroenteritis and Salmonella bacteremia. 

Initial admission

The patient was originally admitted to ARMC for weakness about five weeks ago for 11 days and was found to have gastroenteritis secondary to Salmonella and *E.Coli* with subsequent bacteremia. Chest imaging was deferred upon initial presentation as the patient had a recent CT chest done outpatient for shortness of breath about six weeks before the original admission, which showed multifocal patch diffuse bilateral lung airspace ground-glass opacities unchanged from the previous. He denied any changes in respiratory status upon admission. He was treated with ceftriaxone for nine days, then subsequently with oral cefpodoxime to complete a 14-day course of antibiotics. He had been taking prednisone 10 mg daily and was scheduled for a rituximab infusion one month following discharge for ongoing treatment of microscopic polyangiitis. 

Readmission

The patient was readmitted to ARMC five weeks after the original presentation for about six weeks for ongoing diarrhea and weakness. Upon readmission, the patient was afebrile, tachycardic, and hypoxic, requiring oxygen through a nasal cannula set at 3L. Serum laboratory showed hypokalemia, and the GI BioFire panel (BIOFIRE® FILMARRAY®, BIOMÉRIEUX, France) was positive for *Salmonella enterica*. He was started on intravenous ceftriaxone 2 g daily. Chest X-Ray revealed a new round/cavitary density in the left suprahilar region. Follow-up chest CT without contrast (Figure 1) showed a 3.9-cm cavitary lesion present in the left upper lobe with an air-fluid level, along with diffuse ground-glass opacification involving the lungs bilaterally. The pulmonology service was consulted, with high suspicion that the pulmonary lesion was secondary to ANCA vasculitis. Transthoracic echo was negative for vegetations. Cardiothoracic surgery performed a left robotic thoracoscopy and wedge resection of the left upper lobe cavitary lesion in the second week of the readmission, and the chest tube was removed two days after the surgery.

**Figure 1:**
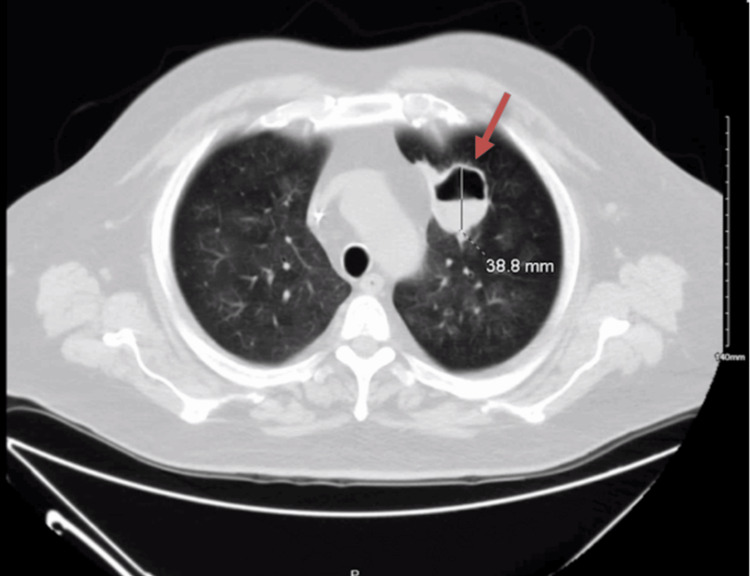
CT chest without contrast The image demonstrates a 3.9-cm cavitary lesion with an air–fluid level in the left upper lobe, along with diffuse bilateral ground-glass opacities.

Pulmonary findings

Biopsy of the cavitation, with subsequent wound culture, revealed acute and chronic fibrosing inflammation, granulation, hemosiderosis, anthracosis, and *Salmonella enteritidis*. Additional fungal and infectious studies were negative, including Strongyloides antibody, Coccidioides antibody, Cryptococcal antigen, Aspergillus antigen, histoplasma antibody, and acid-fast bacilli (AFB) smear X3. 

Treatment and admission complications

Infectious disease was consulted, recommending continuation of intravenous (IV) ceftriaxone 2 g/day for a total of six weeks and atovaquone 1500 mg daily for *Pneumocystis jirovecii* prophylaxis. In the setting of Salmonella bacteremia, a urine culture was obtained for antibiotic susceptibility, confirming sensitivity to ceftriaxone (Table 1). During admission, the patient was found to have native left hip septic arthritis with avascular necrosis and underwent washout with orthopedic surgery (day 28). Per ID recommendations, antibiotic duration was extended with patients completing ceftriaxone 2 g intravenously (IV) daily for four weeks following left hip washout (by that point, the patient had received eight weeks of ceftriaxone). Subsequent imaging revealed deep vein thrombosis of the left popliteal vein, and the patient was started on Eliquis®. Additionally, the patient underwent an infra-red (IR) cryoablation of the right renal mass (day 27), and rituximab therapy was discontinued by oncology/nephrology due to increased risk of infection.

**Table 1:** Antibiotic susceptibility data of Salmonella enterica obtained via urine culture

Urine culture >=1e5 CFU/mL *Salmonella enterica*		
Susceptibility	Minimum Inhibitory Concentration (MIC)	Method not specified
$$ Ampicillin	<8 susceptible	-
$$$ Ampicillin + Sulbactam	<4/2 susceptible	-
$$Cefepime	<2 susceptible	-
Ceftriaxone	<1 susceptible	-
$ Ciprofloxacin	-	Intermediate
$ Trimethaprim + Sulfamethoxazole	<0.5/9.5 susceptible	-

Discharge and follow-up

Upon discharge, the patient was started on cyclophosphamide 750 mg once monthly for six months and subsequently transitioned to mycophenolate mofetil (MMF) 500 mg twice daily (BID) for anti-neutrophil cytoplasmic antibody (ANCA) vasculitis. He was additionally started on amoxicillin for Salmonella infection prophylaxis, given his ongoing immunosuppression and history of disseminated salmonellosis. One month after hospital discharge, the patient returned to the pulmonary outpatient clinic symptomatically well, but with a persistent dry cough. A follow-up imaging five months after surgery revealed resolution of the cavitary lesion (Figure 2), and the patient remains clinically stable. Figure 3 depicts a case progression and outcome flowchart.

**Figure 2:**
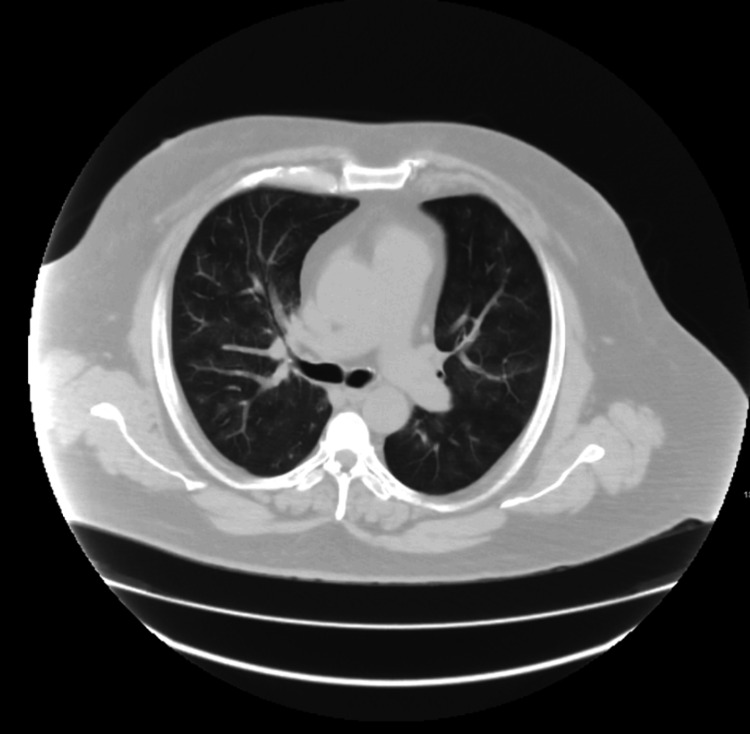
Chest CT image (without contrast) after five months of discharge The image demonstrates resolution of the cavitary lesion in the left upper lobe.

**Figure 3:**
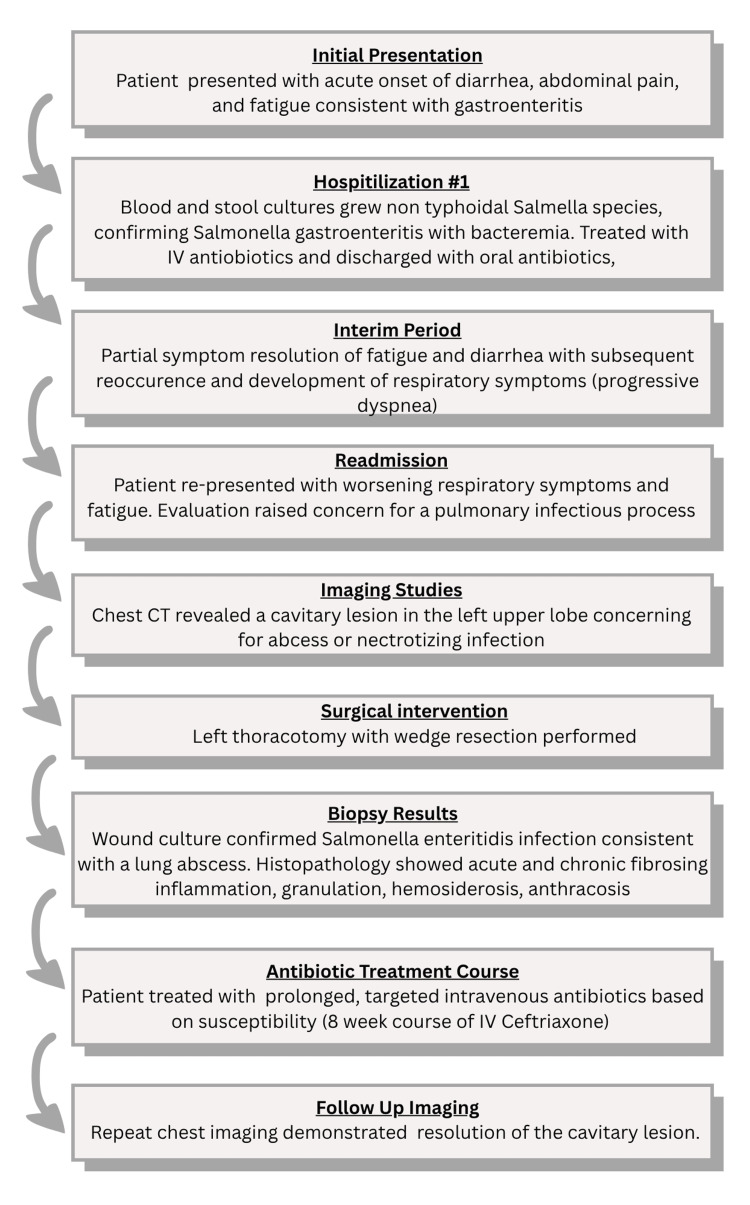
Flowchart depicting case progression and outcome

## Discussion

This is a rare case of Salmonella gastroenteritis manifesting as a cavitary lung lesion in an immunocompromised patient. Despite its immense epidemiological impact and cost to human health, the underlying mechanism by which Salmonella enteritidis crosses the mucosal barrier and subsequently disseminates into the systemic circulation is incompletely understood. Histopathologic assessment of this case provides key insight into the pulmonary environment and the patient’s predisposition to infection. The presence of hemosiderosis suggests previous or ongoing alveolar hemorrhage, which may be associated with underlying ANCA-associated vasculitis. Recurrent microhemorrhaging can compromise normal alveolar macrophage function, producing an environment vulnerable to microbial seeding [5]. Anthracosis, defined by carbon-laden macrophages, demonstrates chronic inhalational exposure (which correlates with the patient's history of tobacco use) and is often associated with impaired mucociliary clearance, further inhibiting natural host defenses [6]. Furthermore, chronic fibrosing inflammation raises concern for underlying interstitial lung disease (ILD). Such remodeling of normal lung architecture would reduce vascular perfusion and impair immune cell migration, leading to increased susceptibility to pulmonary seeding. The combination of such findings suggests that this was not a purely acute infectious process but rather one superimposed on an immunologically compromised pulmonary microenvironment. 

The patient’s immunocompromised state played a key role in the pathogenesis of disseminated Salmonella infection. The use of rituximab, a monoclonal antibody that targets CD20-positive B cells, likely led to prolonged B-cell depletion and impaired humoral immunity, compromising the patient's ability to mount an effective antibody response against encapsulated organisms such as Salmonella [7]. Concomitant corticosteroid use further suppresses both innate and adaptive immunity by inhibiting neutrophil migration, macrophage activation, and T-cell function [8]. Similarly, underlying malignancy impairs cellular immunity and increases systemic inflammation. Together, these variables created an extensive immunocompromised state susceptible to Salmonella invasion.

The presence of a cavitary pulmonary lesion with bilateral ground-glass opacities in an immunocompromised patient prompted a broad differential diagnosis, including infectious, inflammatory, and vasculitic etiologies. ANCA-associated vasculitis was initially considered given the patient’s underlying autoimmune disease and congruent image findings. Pulmonary involvement in ANCA-associated vasculitis may present as nodules, cavitary lesions, or diffuse alveolar hemorrhage (DAH), the latter of which often presents radiographically as bilateral ground-glass opacities. However, the absence of hemoptysis, a significant drop in hemoglobin, and bronchoscopic evidence of hemorrhage made DAH unlikely. The patient’s immunocompromised status in the setting of rituximab therapy and corticosteroid use further expanded the differential to include opportunistic infections. Infections such as aspergillosis commonly present with nodules, cavitation, and ground-glass opacities in immunocompromised hosts. Despite this concern, extensive fungal infectious workup, including serum biomarkers (e.g., galactomannan, β-D-glucan), fungal cultures, and histopathologic evaluation, was negative. Ultimately, a definitive diagnosis was established via tissue sampling, which demonstrated *S. enteritidis* species on tissue culture from the cavitary lung lesion, confirming a pulmonary abscess secondary to disseminated infection. 

In our patient, *S. enteritidis* likely translocated across the intestinal epithelium via M-cell invasion, with subsequent dissemination through the reticuloendothelial system [9]. Salmonella species are known to cause gastroenteritis and bacteremia, but pleural diseases are uncommon [10]. Reported cases show similar findings in immunocompromised individuals with Salmonella in stool cultures, likely resulting in bacteremia that manifests in pulmonary cavitary disease. They are commonly described as single or multiple cavitary lung lesions in an immunosuppressed patient [10,11]. In an immunocompromised individual, differentials of cavitary pneumonia are broad. However, a tissue biopsy after robotic thoracoscopy remains critical for appropriate diagnosis and management. Given our patient’s immunocompromised status (secondary to malignancy, ANCA associated vasculitis and Rituximab use) systemic dissemination of Salmonella most likely occurred via hematogenous spread within the reticuloendothelial system as described above [12]. Of note, hematogenous spread can be transient, leading to negative blood cultures, consistent with our patient’s presentation. As in the case of our patient, many patients experience severe complications (such as joint infections and/or osteomyelitis), reinforcing the importance of early diagnosis and treatment initiation. 

Consistent with the literature, most reported cases of pulmonary *S. enteritidis* infection occur in immunocompromised individuals, including those with predisposing conditions (malignancy, diabetes mellitus, HIV infection) and those receiving immunosuppressive therapies (corticosteroids or biologic agents). Our patient shared many of these risk factors, including malignancy, chronic kidney disease, diabetes, and significant immunosuppression via rituximab and corticosteroid use. Similar to previous case reports, the clinical presentation was nonspecific, with systemic symptoms (weakness, progressive dyspnea) occurring following a diarrheal illness. Radiographically, pulmonary salmonellosis most commonly manifests as solitary or multiple cavitary lesions, frequently involving the upper lobes. Our case aligns with these findings, as the left upper lobe cavitary lesion with an air-fluid level initially raised concern for ANCA-associated vasculitis or opportunistic infection. However, this case is distinguished by several features. First, microbiologic confirmation was obtained via surgical wedge resection rather than more commonly reported sputum or blood cultures, underscoring the diagnostic challenge in such cases. Second, our patient exhibited multifocal dissemination with concurrent septic arthritis, demonstrating a more severe systemic disease burden than often described. Third, the presence of underlying interstitial lung disease and histopathologic findings of chronic lung injury (including hemosiderosis and fibrosis) likely created a permissive environment for focal pulmonary seeding, a factor not commonly emphasized in prior reports.

Treatment strategies reported in the literature generally involve prolonged antibiotic therapy, most commonly third-generation cephalosporins or fluoroquinolones for four to six weeks, with longer durations in cases of abscess or disseminated disease [12]. Our patient required an extended eight-week course of intravenous ceftriaxone due to both pulmonary and extrapulmonary involvement, ultimately resulting in radiographic resolution and clinical stability. This case emphasizes the importance of maintaining a high index of suspicion for *S. enteritidis* as a potential cause of cavitary lung lesions in immunocompromised patients, especially in the setting of recent or concurrent gastrointestinal infection. Table 2 compares a few case reports on Salmonella lung abscess.

**Table 2:** Chart comparing various case reports on Salmonella lung abscess CKD: Chronic kidney disease, DM: Diabetes mellitus, RUL: Right upper lobe, RLL: Right lower lobe, LUL: Left upper lobe, IV: Intravenous.

Study	Host risk factors	Patient presentation	Radiographic findings	Microbiologic diagnosis	Treatment
Nair et al. [10]	Post-operative following esophageal dilation	Fever, productive cough, Progressive dyspnea	Left-sided pleural effusion with consolidation of the left middle and lower lobes	Sputum, pleural fluid, and blood (*S. senftenberg*)	One week on amikacin and ceftriaxone, with subsequent ceftazidime and netilmicin (unspecified length of treatment)
Songkhla et al. [11]	DM Type II, Focal segmental Glomerulosclerosis, Corticosteroid use	Fever, cough	Two cavitary lesions (RUL 2.7 X 2.3 cm and RLL 0.8 X 2.2 cm) and a small right pleural effusion	Sputum culture (Salmonella spp and serogroup B)	Four weeks on IV ceftriaxone followed by eight weeks of oral cefdinir
Present Case	Malignancy, CKD, DM Type II, rituximab therapy, corticosteroid use, vasculitis	Gastroenteritis, weakness, Progressive dyspnea	3.9 cm cavitary lesion (LUL) with an air-fluid level	Surgical tissue	Eight weeks on IV ceftriaxone

Limitations

This report has several limitations. As a single patient case report, the findings are inherently limited in generalizability, and we cannot establish causality between *S. enteritidis* infection and the observed pulmonary pathology. Although tissue cultures confirmed S. enteritidis, the precise route of infection remains uncertain. While hematogenous dissemination is strongly suspected, transient bacteremia limits definitive confirmation of this mechanism. Finally, the absence of standardized treatment guidelines for pulmonary *S. enteritidis* infection limits the ability to compare management strategies. 

## Conclusions

Pulmonary lung abscess is an uncommon manifestation of Salmonella, often presenting in immunocompromised individuals. It is hypothesized that such patients (infected with Salmonella gastroenteritis) undergo hematogenous spread and subsequent spread/seeding of the bacteria. Given Salmonella’s growing antibiotic resistance and the high morbidity/mortality associated with Salmonella septicemia, we hope to increase awareness of Salmonella-associated pulmonary disease, as early detection is essential for timely treatment and for reducing the risk of serious complications.
